# Effects of La-N Co-Doping of BaTiO_3_ on Its Electron-Optical Properties for Photocatalysis: A DFT Study

**DOI:** 10.3390/molecules29102250

**Published:** 2024-05-10

**Authors:** Yang Wang, Qinyan Zhou, Qiankai Zhang, Yuanyang Ren, Kunqi Cui, Chuanhui Cheng, Kai Wu

**Affiliations:** 1 School of Electronics and Information, Xi’an Polytechnic University, Xi’an 710048, China; wangyang@xpu.edu.cn (Y.W.); zhou_qy1031@163.com (Q.Z.); zqk0724@163.com (Q.Z.); cuikunqi_xpu@163.com (K.C.); 2 Xi’an Key Laboratory of Interconnected Sensing and Intelligent Diagnosis for Electrical Equipment, Xi’an Polytechnic University, Xi’an 710048, China; 3 State Key Laboratory of Electrical Insulation and Power Equipment, Xi’an Jiaotong University, Xi’an 710049, China; wukai@xjtu.edu.cn; 4 Electric Power Research Institute of Yunnan Power Grid Company Ltd., China Southern Power Grid, Kunming 650217, China; 5 Electric Power Research Institute, China Southern Power Grid, Guangzhou 510663, China

**Keywords:** BaTiO_3_, DFT, photocatalysis, light absorption, band structure

## Abstract

In cation–anion co-doping, rare earth elements excel at regulating the electronic structure of perovskites, leading to their improved photocatalytic performance. In this regard, the impact of co-doping rare earth elements at the Ba and Ti sites in BaTiO_3_ on its electronic and photocatalytic properties was thoroughly investigated based on 2 × 2 × 2 supercell structures of BaTiO_3_ with different La concentrations of 12.5% and 25% using DFT calculations. The band structure, density of states, charge density difference, optical properties, and the redox band edge of the co-doped models mentioned above were analyzed. The results indicated that the BaTiO_3_ structure co-doped with 25% La at the Ti site exhibited higher absorption in the visible range and displayed a remarkable photocatalytic water-splitting performance. The introduced La dopant at the Ti site effectively reduced the energy required for electronic transitions by introducing intermediate energy levels within the bandgap. Our calculations and findings of this study provide theoretical support and reliable predictions for the exploration of BaTiO_3_ perovskites with superior photocatalytic performances.

## 1. Introduction

Various new energy solutions have been developed to address and overcome energy and environmental issues [1]. Among these, hydrogen production through semiconductor photocatalysis is considered to be one of the most promising pathways for producing green energy [2,3]. Due to their excellent photocatalytic activity, perovskite oxides (ABO_3_) are considered to be excellent candidates for the photocatalytic splitting of water and the production of hydrogen [4,5]. In the family of perovskite oxides, BaTiO_3_ has attracted considerable attention due to its remarkable ability to catalyze water decomposition reactions. However, this photocatalyst has a relatively wide bandgap of 3.20 eV, which allows for the absorption of solar light only within the ultraviolet region (constituting approximately 7% of the total solar energy). This imposes certain limitations on light absorption in water-splitting applications [6,7]. Therefore, there is an urgent need to find appropriate methods to modulate its bandgap energy in order to achieve the required balance between its light absorption capability and water-splitting performance.

Researchers have suggested numerous approaches in an effort to lower the bandgap width of perovskite and increase its catalytic activity within the visible light spectrum. One of the most direct and effective methods is to dope external elements into the perovskite catalysts. The earliest method introduced was mono-doping, which can be classified into anion doping and cation doping. F-doped BaTiO_3_ exhibits an enhanced photocatalytic activity as a result of the reduced recombination of photogenerated electrons and holes hindered by the F ions doped in the material [8]. Experimental studies have also shown that N-doped BaTiO_3_ leads to a reduction in its bandgap by introducing oxygen vacancies inside its structure, thereby enhancing its light absorption efficiency in the visible range [9]. The density functional theory (DFT) was also employed as a means of explaining the physical mechanisms underlying the enhancement of visible light absorption by non-metal element doping. Teng et al. used DFT calculations to study the effects of B, C, and N on BaTiO_3_’s electronic and optical properties. The incorporation of B and N elements into the BaTiO_3_ lattice results in impurity states near the Fermi level, causing a reduction in the bandgap. Conversely, the presence of carbon element results in the replacement of both the valence band maximum (VBM) and the conduction band minimum (VBM) in BaTiO_3_, which lowers its bandgap energy [10]. Modifications in the electronic and optical properties of SrTiO_3_ doped with N and S were also analyzed and compared by Rehman et al. A tiny bandgap formed by N-2p and O-2p orbitals was discovered in the N-doped SrTiO_3_, which could lead to enhanced electron–hole separation and facilitate light absorption [11]. The anion doping research discussed above primarily aimed to reduce the bandgap of a catalyst by altering its valence band structure.

Numerous studies have also been dedicated to improving the photocatalytic properties of perovskite through metal cation doping. An enhancement in the absorption of visible light has been observed in BaTiO_3_ samples doped with Mo, which results in a notable increase in the efficiency of hydrogen production. The reduction in CBM caused by the coupling of Ti-3d and Mo-3d orbitals was found to be attributed to the enhancement of its photocatalytic activity [12]. According to Issam et al., the substitution of Cr at Ti sites creates defect states near the Fermi level, which serve as intermediate states facilitating the electron transfer from VBM to CBM, thus enhancing its visual light absorption properties in Cr-doped BaTiO_3_ [13]. A comparison of the effects of Sr, Zn, Nd, Ni, Zr, and Hf doping at the Ba and Ti sites of BaTiO_3_ has indicated a reduction in the bandgap when Sr, Zn, and Nd are doped at the Ba site. Specifically, electron overlap near the Fermi level was observed when Nd was used as the dopant [14]. The introduction of metal cations at the Ba site of perovskite structures can influence the bandgap and optical absorption properties by adjusting the electronic energy levels of the material. However, Ti^4+^ may transform into Ti^3+^ when metal elements are doped at the Ti site, thus creating oxygen vacancy defect states that could affect the conductivity and the overall electron-optical performance of the material. In general, the doping of metal elements leads to changes in the CBM or introduces intermediate impurity states within the bandgap, thereby influencing the photocatalytic activity [15]. Rare earth elements have gradually been recognized for their potential application as dopants to improve perovskite’s photocatalytic properties. Through defect state calculations, Yang et al. found that rare earth element doping, especially La element doping, is a promising approach for enhancing the optical performance of perovskites [16]. Despite the positive results of the cation or anion doping in improving the photocatalytic properties of perovskites, it is important to note that inappropriate doping may result in lattice defects that can decrease the structural stability of the photocatalytic materials. Additionally, the photocatalytic activity may also be adversely affected by these imperfections, as they encourage the recombination of electrons and holes generated by photosynthesis. Therefore, careful consideration and optimization of doping approaches are necessary to reduce the potential side effects of doping strategies when improving the photocatalytic activity of perovskites.

A co-doping strategy has gradually come into focus in attempts to minimize lattice-induced side effects while precisely controlling the electronic structure of BaTiO_3_. Co-doping is mainly divided into three categories: cation–cation co-doping [17,18,19], anion–anion co-doping [20,21,22], and cation–anion co-doping [23,24,25]. For example, compared to undoped BaTiO_3_, those co-doped with Fe^3+^ and Cr^3+^ have been shown to exhibit a redshift in the absorption edge owing to the adjustment of cations [26]. SrTiO_3_ perovskites were co-doped with La and Al dopants to improve their photocatalytic performance by suppressing the formation of defects such as oxygen vacancies and Ti^3+^ in the SrTiO_3_ perovskite lattice [27]. In terms of anion–anion co-doping, F and N were used to influence the lattice structure of TiO_2_ to enhance its photocatalytic efficiency [28]. Unoccupied or occupied local states can be controlled between the valence and conduction bands through the appropriate incorporation of the anion dopant, which is the fundamental mechanism responsible for re-confining the bandgap energy [29]. Nevertheless, the co-doping of cations or anions can only be used to control the positions of the valence and conduction bands independently, resulting in some limitations in the overcall adjustment of the band structure. By contrast, cation–anion co-doping involves adjusting the CBM using cations and the CBM using anions, respectively, and introducing impurity energy levels into the bandgap, which is a synergistic band structure regulation. Furthermore, it reduces the generation of lattice defects, enhancing the stability of perovskites [30]. For example, DFT calculations on Zr-(S/Se/Te) co-doped BaTiO_3_ demonstrate that this kind of cation–anion co-doped perovskite reduced the bandgap energy and enhanced the thermodynamic stability [31]. The rare earth elements are also used in co-doping approaches to improve the photocatalytic performance of perovskite. La-N co-doped perovskite was investigated by Miyauchi et al. in order to improve the photocatalytic efficiency [32]. In the study by Zhang et al. focusing on co-doped SrTiO_3_ with (La/Ce/Pr) and N, there was no single level of donor or acceptor, so the electrons from the rare earth donor level (La/Ce/Pr) passivated the same number of holes on the N acceptor level [33]. Furthermore, Wang et al. effectively reduced the bandgap of SrTiO_3_ using the co-doping combinations of N–La, C–Ce, and N–Ce–N. Compared to mono-doped models, these co-doped models showed a superior performance in the photocatalytic efficiency, with a reduced bandgap and charge carrier recombination centers [34].

According to the discussion above, rare earth elements exhibit considerable potential for regulating the electronic structure of perovskites to improve their photocatalytic performance, and cation–anion co-doping constitutes a synergistic method of doing so. To our knowledge, however, little research has been conducted on perovskites that are co-doped with rare earth elements and nitrogen, both experimentally and theoretically. We have, therefore, conducted an in-depth analysis of the impacts of different doping sites and dopant concentrations on the electronic structure and photocatalytic properties of La-N co-doped BaTiO_3_ using DFT calculations. This research aims to clarify the underlying mechanisms of the enhanced photocatalytic properties of perovskites when rare earth elements and non-metal elements are co-doped as well as provide insight into the design of new perovskites catalysts based on band structure optimization.

## 2. Results and Discussion

### 2.1. Mono-Doped BaTiO_3_ with La or N

#### 2.1.1. Atomic Structure and Formation Energy

The optimized lattice parameters obtained for pure BaTiO_3_ (a = b = c = 4.028 Å) were consistent with experimental data (a = b = c = 3.996 Å [35]), as well as with theoretically calculated values (a = b = c = 4.030 Å [36]). The distance between Ba and its closest O atoms was found to be 2.848 Å, while the predicted Ti-O bond length was computed as 2.014 Å. These values agreed well with the experimental values of 2.008 Å and 2.839 Å, respectively [37]. This suggested that the simulated model of BaTiO_3_ with the parameter values that were employed was quite appropriate for further computational studies.

The formation energies ($E_{f}$) for different mono-doped models were calculated using Equation (1), where these formation energies served as a measure of the structural stability [33].

(1) $$E_{f}=(E_{\mathrm{doped}}-n_{\mathrm{doped}}\mu_{\mathrm{doped}})-(E_{\mathrm{pure}}-n_{\mathrm{subsituted}}\mu_{\mathrm{subsituted}})$$

Here $E_{\mathrm{doped}}$ denotes the total energy of the BaTiO_3_ mono-doped or co-doped model, $E_{\mathrm{pure}}$ represents the total energy of the BaTiO_3_ undoped model, whereas $n_{\mathrm{doped}}$ and $n_{\mathrm{substitute}}$ represent the number of doped elements and the number of replaced elements, respectively. $\mu_{\mathrm{doped}}$ and $\mu_{\mathrm{substitute}}$ are the chemical potential of the doped element and the replaced element. In this study, the values of $\mu_{\mathrm{Ba}}$, $\mu_{\mathrm{Ti}}$, and $\mu_{\mathrm{La}}$ were obtained from the energy of each atom in the respective block crystal, whereas the values of $\mu_{O}$ and $\mu_{N}$ were calculated from an O or N atom in the O_2_ or N_2_ molecule placed at the center of a 10 × 10 × 10 cubic box. Since the chemical potential fluctuates during doping, we calculated it separately for the Ti-rich and Ti-poor cases, as depicted in Equation (2) [38]:

(2) $$\mu_{\mathrm{BaTi}O_{3}}-\mu_{\mathrm{Ba}-\mathrm{bulk}}-3\mu_{O-\mathrm{gas}}\leq \mu_{\mathrm{Ti}}\leq \mu_{\mathrm{Ti}-\mathrm{bulk}}$$

where $\mu_{\mathrm{BaTi}O_{3}}$ represents the total energy of pristine BaTiO_3_. The upper and lower limits of $\mu_{\mathrm{Ti}}$ are regarded as the Ti-rich and Ti-poor cases. By inserting the two calculated values into Equation (1), the formation energy for both the Ti-rich and Ti-poor cases can be determined.

**Figure 1:**
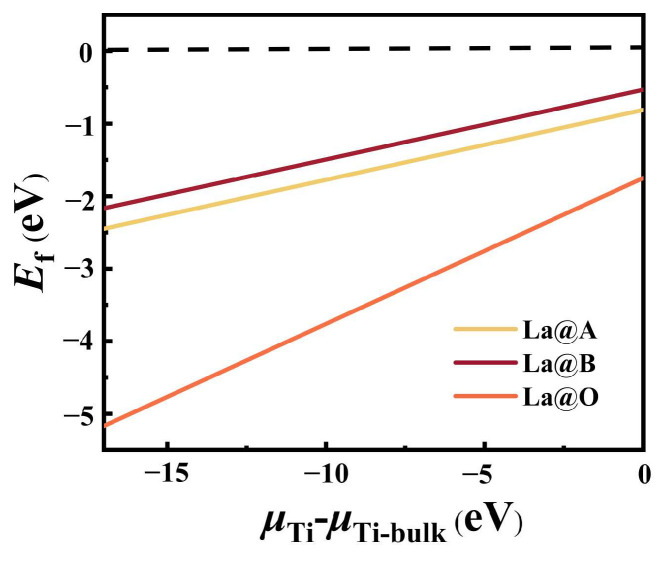
The defect energies of different mono-doping models with variations in Ti’s chemical potentials.

The integration of the dopant into the lattice becomes more beneficial if it leads to a more negative defect formation energy in terms of the structural stability. Figure 1 shows the calculated defect energy of different mono-doped models with variations in Ti’s chemical potential [39]. In both Ti-poor and Ti-rich cases, the formation energies for all mono-doped models were negative, indicating the rationality of the mono-doped BaTiO_3_ models we proposed. The observation of a lower formation energy in the case where N replaced O and the obtained formation energy values indicated that this substitution was the most advantageous. This can primarily be attributed to the fact that O^2−^ (1.40 Å) and N^2−^ (1.71 Å) exhibited similar ionic radii. Additionally, a comparison of the mono-doped models of La suggested that La is more likely to substitute the Ba site. This tendency may be ascribed to the larger difference between the ionic radii of La^3+^ and Ti^3+^ (1.06 Å vs. 0.61 Å) compared to the difference between the ionic radii of La^3+^ and Ba^2+^ (1.06 Å vs. 1.35 Å), making it easier for La to replace Ba within the lattice structure.

#### 2.1.2. Electronic Structure

**Figure 2:**
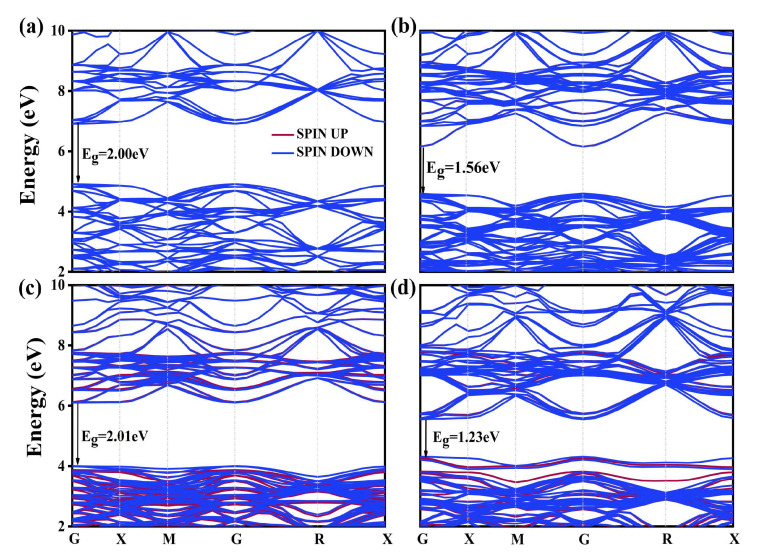
Band structure of the (**a**) pristine BaTiO_3_, (**b**) La@A-doped BaTiO_3_, (**c**) La@B-doped BaTiO_3_, and (**d**) N@O-doped BaTiO_3_.

The effects of La and N mono-doping on the electronic structure of BaTiO_3_ were analyzed by first computing the band structures and electronic density of states (DOS) of the mono-doped models and then comparing them with the results obtained for the pristine BaTiO_3_ model. The Ba-1s core energy level was used as the reference energy to align the band structures and DOS for different doping models of BaTiO_3_. The computed band structures are displayed in Figure 2. The band structure of pristine BaTiO_3_ is presented in Figure 2a. The unavoidability of bandgap underestimation in DFT calculations is well documented. The bandgap of pristine BaTiO_3_ in this study was estimated using the GGA + U technique, and its value was obtained to be 2.00 eV, which was still 1.20 eV lower than its experimental value. However, in comparison to previous computational studies, the band structure properties and gap shifts were still quite reliable [40,41,42].

The remaining plots in Figure 2 illustrate the influence of La doping at the Ba and Ti sites, along with N doping at the O site, on the band structure of BaTiO_3_. All mono-doped structures displayed direct bandgap characteristics, and no single donor or acceptor energies were observed in the forbidden region between the conduction band (CB) and valence band (VB). Both the CB and VB moved downwards in energy as La replaced the Ba site, resulting in a reduced bandgap energy of 1.55 eV. In comparison to the pristine model, no significant change in the band structure was observed as La replaced the Ti site, with only a slight increase of 0.10 eV in the bandgap energy. Finally, the overall CBM shifted to lower energy levels for the N mono-doped model, leading to a decreased bandgap. This resulted in a bandgap energy of 1.23 eV for this doped model. 

**Figure 3:**
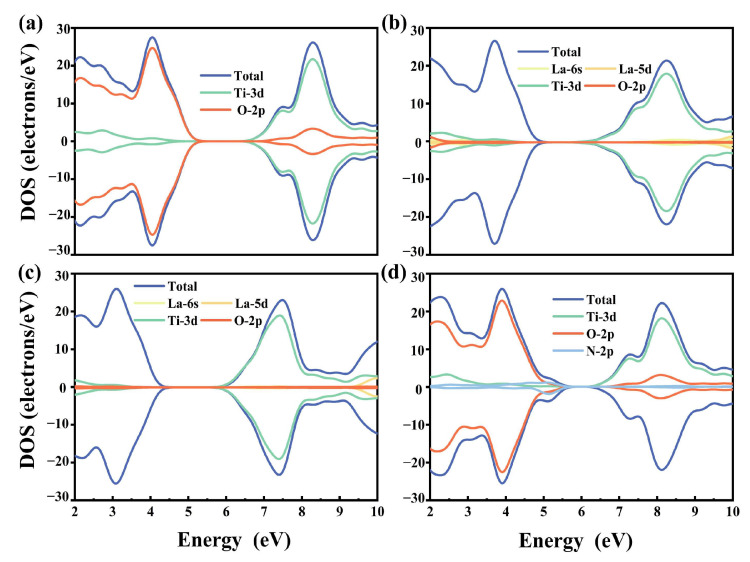
The TDOS and PDOS correspond to different orbitals for (**a**) pristine BaTiO_3_, (**b**) La@A-doped BaTiO_3_, (**c**) La@B-doped BaTiO_3_, and (**d**) N@O-doped BaTiO_3_.

The plots for the calculated partial density of states (PDOS) and total density of states (TDOS) are provided in Figure 3. The results showed that most of the valence band minimum (VBM) and conduction band maximum (CBM) in pristine BaTiO_3_ were made up of Ti-3d and O-2p orbitals. Conversely, Ba did not contribute to the VBM or CBM, even though it provided electrons to maintain the charge balance within the model. Thus, the bandgap of BaTiO_3_ was determined by the respective energy positions of the Ti-3d and O-2p orbitals, as illustrated in Figure 3a. These results were quite consistent with earlier computations [43].

The contribution of the O-2p orbitals to the VB greatly diminished in the La-doped model, while the Ti-3d states continued to dominate the composition of the CB. The relative edge positions of the VBM and CBM were not significantly affected by the substitution of La in the position of Ti, with only a 0.09 eV increase in the bandgap energy. This suggested that La mono-doping at the Ti site was not an ideal choice for broadening the semiconductor absorption edge. In contrast, the substitution of La at the Ba site led to an overall downward shift of the conduction and valence band positions, simultaneously reducing the distance between the CBM and VBM. The introduction of N-2p states caused the VBM to extend to higher energies, while the CBM moved to lower energies, effectively narrowing the bandgap. These findings underscore the subtle modulation of the electronic structure by the mono-doping of La and N, providing a theoretical foundation for subsequent optimization strategies in co-doping.

**Figure 4:**
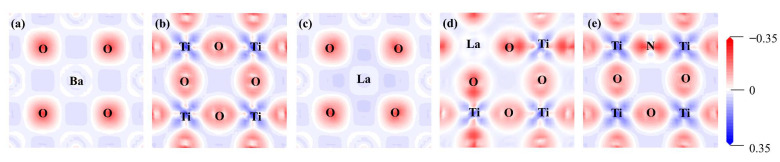
The charge density difference for the (**a**) pristine BaTiO_3_ (1 0 0), (**b**) pristine BaTiO_3_ (0 1 0), (**c**) La@A-doped BaTiO_3_, (**d**) La@B-doped BaTiO_3_, and (**e**) N@O-doped BaTiO_3_. An iso-surface value of 0.1 e/Å ^3^ was used, with red and blue clouds indicating the accumulation and depletion of negative charges (electrons), respectively.

Due to the difference in doping sites, the charge density difference of (1 0 0) and (0 1 0) crystal planes were computed for the pristine and mono-doped BaTiO_3_ models, as shown in Figure 4. A comparison between Figure 4a,c reveals that the degree of charge transfer between La and O is greater than that between Ba and O when La is doped at Ba site. When La was doped at the Ti site, there was a significant decrease in the amount of charge transfer between La and O, as well as between other Ti and O atoms. Additionally, the results presented in Figure 4e demonstrate that the introduction of N enhances the charge transfer between Ti and N, as well as between other Ti and O atoms.

The charge density difference can only provide qualitative information about charge transfer between atoms. The Bader atomic charge was then calculated to quantify the electron gain and loss at each atom in all BaTiO_3_ doping models [44]. The Bader atomic charge values for each atom were calculated by subtracting the valence electrons of each atom from the total charge; the average values for each type of atom in different BaTiO_3_ models are shown in Table 1. OPT in the table represents the pristine BaTiO_3_. Meanwhile, the atomic Bader charge values of each atom for pristine and mono-doped BaTiO_3_ models are shown in Table S1 in the Supplementary Materials, which quantifies the atomic charges on each atom. The charge transfer direction and quantity could be systematically illustrated by comparing the charge variations before and after doping, which is in accordance with our charge density difference diagram in Figure 4. In the pristine BaTiO_3_ model, Ba and Ti donated electrons, forming ionic and covalent bonds with O, respectively. This was consistent with the results of the charge density-difference maps shown in Figure 4a,b. In the La@A model, the number of electrons lost by La exceeded that for Ba, explaining the observed higher La-O bond strength, as shown in Figure 4c. Upon the introduction of N at the O site, a slight variation in the atomic losses around the Ti atoms was observed in comparison to the pristine BaTiO_3_ model, along with a minimal overall change. Additionally, the Bader atomic charge at the N site was found to be slightly lower in comparison to that at the O site, owing to its lower electronegativity and larger atomic radius. Furthermore, in the pristine BaTiO_3_ model, the distribution of electronic gains and losses among the three types of atoms was uniform, with Bader atomic charge values for Ba, Ti, and O being 1.57, 2.17, and −1.25, respectively. These mono-doped models exhibited only minor alterations in their electronic distributions for each atom.

**Figure 5:**
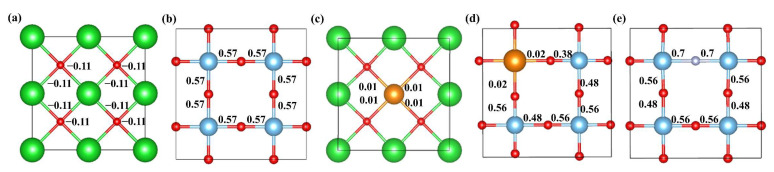
The Mulliken bond populations for the (**a**) pristine BaTiO_3_ (1 0 0), (**b**) pristine BaTiO_3_ (0 1 0), (**c**) La@A-doped BaTiO_3_ (1 0 0), (**d**) La@B-doped BaTiO_3_ (0 1 0), and (**e**) N@O-doped BaTiO_3_ (0 1 0). Ba atoms are shown in green, Ti atoms in blue, O atoms in red, La atoms in orange, and N atoms in grey.

The bonding between different atoms inside BaTiO_3_ before and after doping were investigated by Mulliken bond population calculations [45], which quantify the extent of electron sharing between two atoms, providing deep insights into the bonding characteristics of perovskite materials [46]. It should be noted, however, that negative values for Mulliken bond populations are possible, indicating that the bond type is ionic and relatively easy to break if the absolute value is low [47]. Mulliken bond populations do not have a precise threshold value for distinguishing between ionic and covalent bonds, but a bond with a Mulliken bond population greater than 0.1 is normally considered covalent, according to the literature [48,49,50]. Mulliken bond populations were calculated for five cut planes depicting typical bonds in pristine and doped BaTiO_3_, as shown in Figure 5. In Figure 5a, a small negative value of −0.11 was found for all Ba-O bonds in pristine BaTiO_3_, indicating that an ionic bond was formed between Ba and O. The introduction of La atoms into BaTiO_3_ results in La ionic bonds forming between La and O in both La@A and La@B models, because La-O bond populations are 0.01. In contrast, Ti atoms formed covalent bonds with O and N atoms in both pristine and doped models.

#### 2.1.3. Water Redox Potential

**Figure 6:**
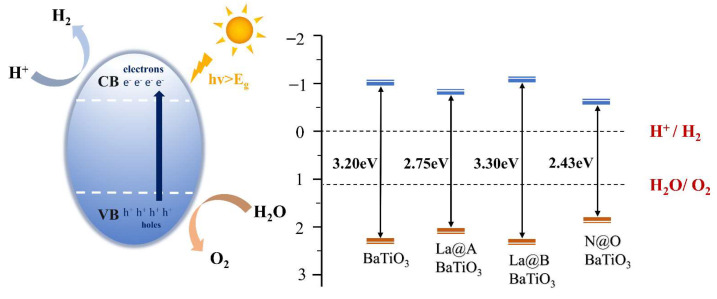
Band edge arrangement of pristine BaTiO_3_ and the mono-doped BaTiO_3_ models in reference to the potential at the edge of the water redox zone.

The CBM energies ($E_{\mathrm{CB}}$) and VBM energies ($E_{\mathrm{VB}}$) were empirically calculated according to Equation (3) in order to describe the oxidation and reduction capacities of La and N mono-doped BaTiO_3_ models [51]:

(3) $$E_{\mathrm{CB}}=X-\frac{1}{2}E_{g}-E_{0}$$

where $E_{g}$ is the bandgap value after adding the “scissor” operator, X is the absolute electronegativity of perovskite oxide, and $E_{0}$ is the energy of free electrons relative to the normal hydrogen electrode (~4.5 eV). It is commonly recognized that the location of the band edges in relation to the redox potentials controls the photocatalytic capacity of the semiconductors, allowing them to split water and produce hydrogen. To achieve the water-splitting reaction, the VBM must be located below the oxygen reduction level (O_2_/H_2_O), and the CBM must be located above the hydrogen evolution level (H^+^/H_2_). The photocatalytic efficiency increases as the CBM gets closer to the H^+^/H_2_ reduction level. The obtained data are visualized in Figure 6.

The CBM of pristine BaTiO_3_ was positioned 0.970 eV above the hydrogen reduction (H^+^/H_2_) level, while the corresponding VBM was located 2.230 eV below the water oxidation (O_2_/H_2_O) level, consistent with recent research findings [52]. For the La@A, La@B, and N@O models, the $E_{CB}$ values were calculated to be −0.75 eV, −1.02 eV, and −0.59 eV, respectively. The corresponding $E_{VB}$ values were determined to be 2.16 eV, 2.28 eV, and 1.85 eV, respectively. The band edge positions for all mono-doped BaTiO_3_ models satisfied the requirements for water redox reactions, indicating their thermodynamic capability to facilitate water decomposition. The La@A BaTiO_3_ model slightly allowed the CBM and VBM to come closer to the redox level. Conversely, the band edges moved further away from the water oxidation and reduction levels in the La@B BaTiO_3_ model. On the other hand, the N@O model enhanced the closeness between the CBM and the H^+^/H_2_ levels. Therefore, in the absence of considering its efficiency, N doping may be the optimal choice among mono-doping strategies to enhance photocatalytic activity.

### 2.2. La-N Co-Doped BaTiO_3_

#### 2.2.1. Formation Energy and Binding Energy

**Figure 7:**
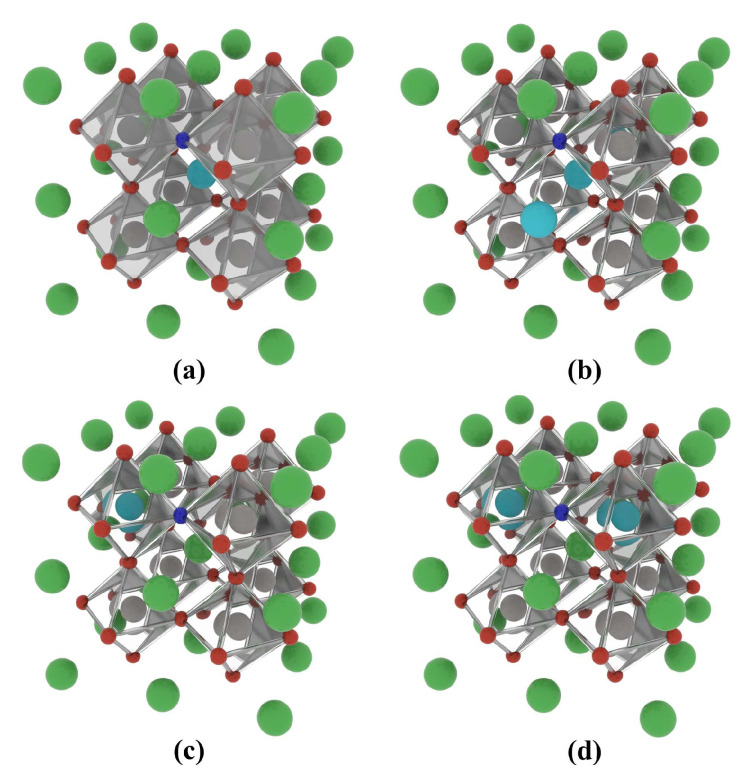
Co-doped atomic models of the BaTiO_3_ crystal based a 2 × 2 × 2 supercell: (**a**) 12.5% La-N@A co-doping, (**b**) 25% La-N@A co-doping, (**c**) 12.5% La-N@B co-doping, and (**d**) 25% La-N@B co-doping. Ba atoms are shown in green, Ti atoms in grey, O atoms in red, La atoms in mint blue, and N atoms in dark blue.

The La-N co-doped BaTiO_3_ atomic structures that were simulated and studied are shown in Figure 7. The models are as follows: 12.5% La and N co-doped at the Ba site, 25% La and N co-doped at the Ba site, 12.5% La and N co-doped at the Ti site, and 25% La and N co-doped at the Ti site. For ease of reference in the following text, these models are denoted as 12.5% La-N@A, 25% La-N@A, 12.5% La-N@B, and 25% La-N@B, respectively.

The formation energies for La-N co-doping at different sites and concentrations were calculated in a manner similar to the mono-doping cases, with some modifications to the formula. The modified formula is as follows: [38]

(4) $$E_{f}=(E_{\mathrm{doped}}-\sum n_{\mathrm{doped}}\mu_{\mathrm{doped}})-(E_{\mathrm{pure}}-\sum n_{\mathrm{substituted}}\mu_{\mathrm{substituted}})$$

where $n_{\mathrm{doped}}$ and $n_{\mathrm{substitute}}$ represent the number of doped elements and the number of replaced elements, respectively. Similarly, the two calculated values obtained from Equation (2) can be substituted into Equation (4) to determine the formation energies for Ti-rich and Ti-poor models.

**Figure 8:**
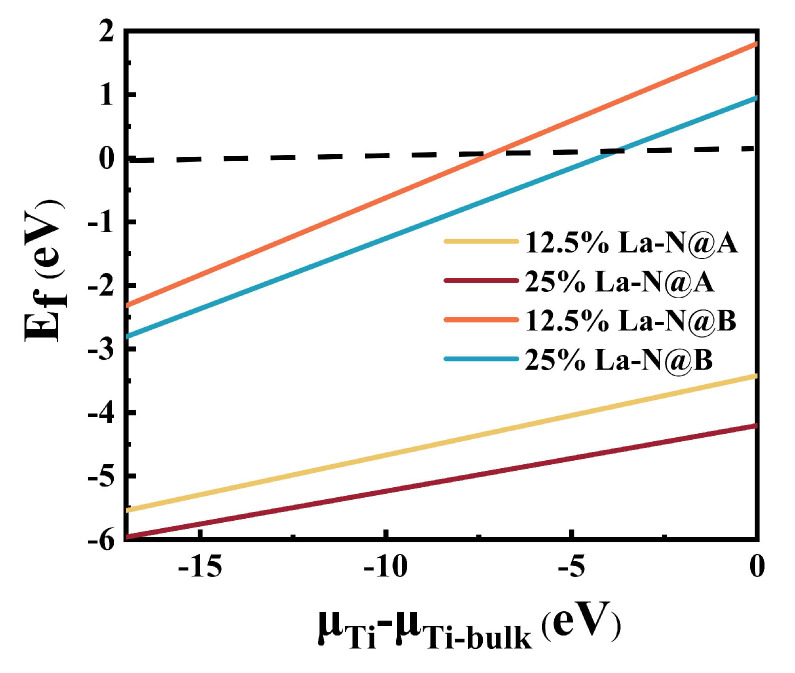
The defect energies of different co-doping models with variations in Ti’s chemical potentials.

It can be observed from Figure 8 that the defect energies of the co-doped models increase with the chemical potential of Ti, indicating that the Ti-poor condition is more favorable for the growth of these defects. Regardless of the Ti concentrations, the defect energies for La doping at the A site of BaTiO_3_ models are negative and decrease in magnitude with an increase in the La doping concentration, suggesting a more stable structure for La-N@A co-doped models. Conversely, when La is doped at the B site, the defect energies are greater than 0 for both La doping concentrations in Ti-rich models. La-N co-doped BaTiO_3_ models with lower concentrations of La are more difficult to form than those with higher concentrations of La. In other words, La doping at the B site for La-N co-doped BaTiO_3_ models in Ti-rich conditions is difficult to achieve experimentally.

The defect binding energy ($E_{b}$) was calculated using the following equation to assess the coupling strengths of the dopants in the co-doped models [53]:

(5) $$E_{b}=E_{\mathrm{La-doped}}+E_{\mathrm{N-doped}}-E_{\mathrm{opt}}-E_{\mathrm{co-doped}}$$

where $E_{\mathrm{La-doped}}$, $E_{\mathrm{N-doped}}$, $E_{\mathrm{co-doped}}$, and $E_{\mathrm{opt}}$ are the total energies of La mono-doped BaTiO_3_, N mono-doped BaTiO_3_, La-N co-doped BaTiO_3_, and pristine BaTiO_3_, respectively.

The calculated binding energies for the co-doped models are summarized in Table 2. A positive $E_{b}$ value indicates that the defects are stable relative to individual defects. The data in the table reveal that the La-N@A co-doped model is stable, with its stability decreasing with an increase in the La concentration. Conversely, the defect energies calculated for the La-N@B co-doped model were all negative, indicating that it exhibited a lower stability in comparison to the mono-doped models and that the ability of defects to bind was weaker for this model. From this perspective, the BaTiO_3_ model with La co-doping and with N at the Ba site was relatively more stable.

#### 2.2.2. Electronic Structure

**Figure 9:**
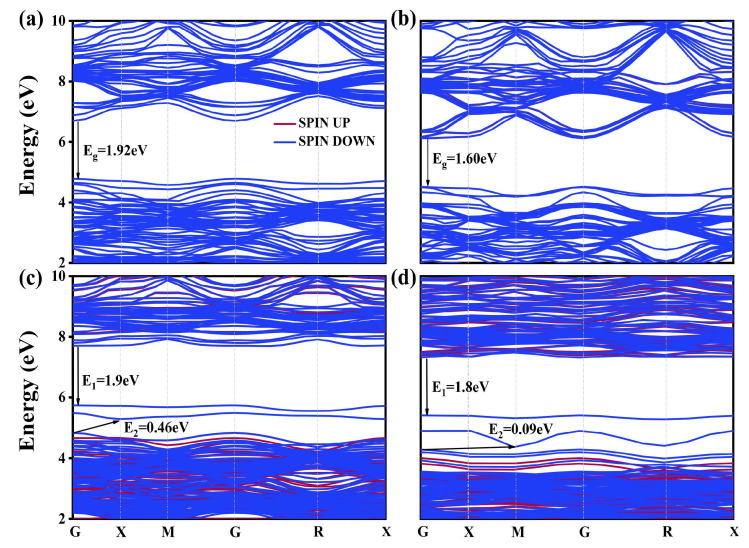
Band structure of the (**a**) 12.5% La +N@A co-doped BaTiO_3_, (**b**) 25% La-N @A co-doped BaTiO_3_, (**c**) 12.5% La-N@B co-doped BaTiO_3_, and (**d**) 25% La-N@B co-doped BaTiO_3_.

**Figure 10:**
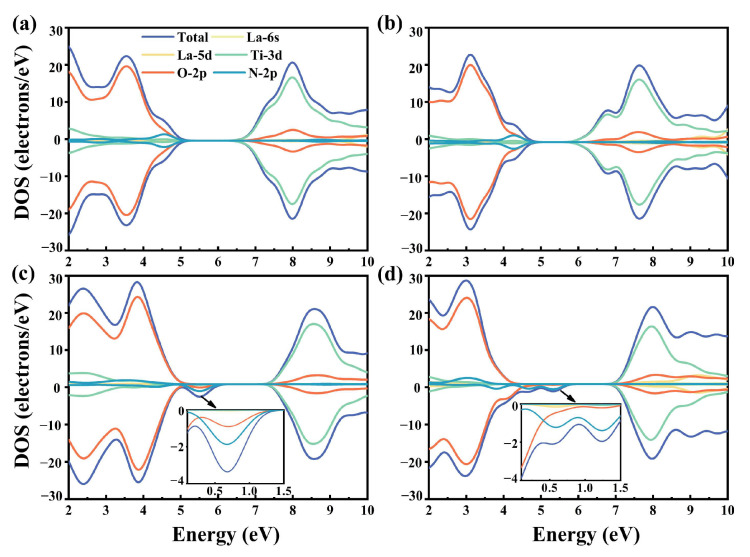
The TDOS and PDOS correspond to different orbitals for (**a**) 12.5% La-N@A co-doped BaTiO_3_, (**b**) 25% La-N @A co-doped BaTiO_3_, (**c**) 12.5% La-N@B co-doped BaTiO_3_, and (**d**) 25% La-N@B co-doped BaTiO_3_.

The obtained band structures and DOS for these models are presented in Figure 9 and Figure 10, respectively. The figure shows that BaTiO_3_ co-doped with 12.5% and 25% La-N@A exhibits characteristics of a direct bandgap semiconductor, while BaTiO_3_ co-doped with the same concentrations of La-N at the Ti site displays characteristics of an indirect bandgap semiconductor. For the model of La-N@A co-doping of BaTiO_3_ (as shown in Figure 9a,b, it was observed that there were no intermediate energy levels between the conduction and valence bands. This indicated electron passivation between La that provided the donor-level electron and N that offered the acceptor-level hole [33]. The positions of CB and VB remained largely unchanged as one La atom substituted a single Ba atom, while the bandgap reduced slightly by 0.08 eV. However, the CBM and VBM were shown to move downward overall as the La concentration was increased, which further reduced the bandgap to 1.62 eV.

The introduction of intermediate impurity states in the forbidden region between the CB and VB, as a result of Ti-site co-doping, can be seen clearly in Figure 9c,d. The introduction of La^3+^ ions at the Ti site is primarily responsible for the creation of intermediate bands (IBs). In addition, the excess electrons offered by the doping atoms (La) contribute to the formation of N^3+^ ions with stable outermost electron structures [54,55]. Conversely, the lack of electrons led to the formation of acceptor levels, which corresponded to intermediate states when La^3+^ ions replaced Ti^4+^ ions. These transitional states can function as intermediate levels for electrons while moving from the VB to the CB during the photocatalysis process. These intermediate states provide two steps near the conduction band and valence band to help the electrons jump from the VBM to the bottom of the intermediate state or from the top of the intermediate state to the CBM. We labeled these distances as *E*_1_ and *E*_2_. The *E*_1_ values for the two co-doping models were calculated to be 0.46 eV and 0.09 eV, respectively. Their *E*_2_ values were determined to be 1.9 eV and 1.8 eV, respectively. The magnitude of *E*_1_ increased with an increase in the La concentration, whereas no notable variation was observed in the value of *E*_2_. The presence of intermediate states enhanced optical absorption and served as recombination centers for photogenerated charge carriers, promoting their recombination. However, it is important to note that the intermediate bandgap shown in the Figure 9c,d is the unoccupied intermediate states. This partially occupied intermediate state may serve as a site of recombination for charge carriers produced by photosynthesis, thereby leading to a reduction in the photoelectrochemical efficiency of the semiconductor [56,57,58].

The results presented in Figure 10a,b clearly show that the incoming dopant atom La does not make any direct impact on the band structure of BaTiO_3_ when it replaces a Ba atom, since the substituted Ba atom exerts minimal influence on the CB and VB. On the other hand, it was observed that the influence of La-5d orbitals on the upper section of the CB gradually became more prominent with an increase in the concentration of the La dopant atoms. Simultaneously, the CB and VB moved towards the lower energy level. In contrast, an intermediate state was introduced between the conduction and valence bands for La atoms co-doped at Ti sites, which was closer to the VB. The enlarged images of relevant regions are provided in Figure 10c,d to clearly show the contributions of different electronic orbitals to the intermediate state, the arrows in the figure indicate pointing to the enlarged image. The intermediate state created within the 12.5% La-N co-doped BaTiO_3_ model was mostly generated by the hybridization of the N-2p and O-2p orbitals, while the VBM was still primarily composed of the O-2p orbitals. The O-2p-orbital-dominated VBM extended towards higher energies in the 25% La-N co-doped BaTiO_3_ model, while the main part of the intermediate state was composed of the N-2p orbitals, and the contribution of the O-2p orbitals gradually decreased. Additionally, the contribution of La-5d states to the Ti-3d states slightly increased, possibly owing to the substitution of the Ti atoms by La atoms, which was a major contributor to the CB of the pristine BaTiO_3_ model.

**Figure 11:**
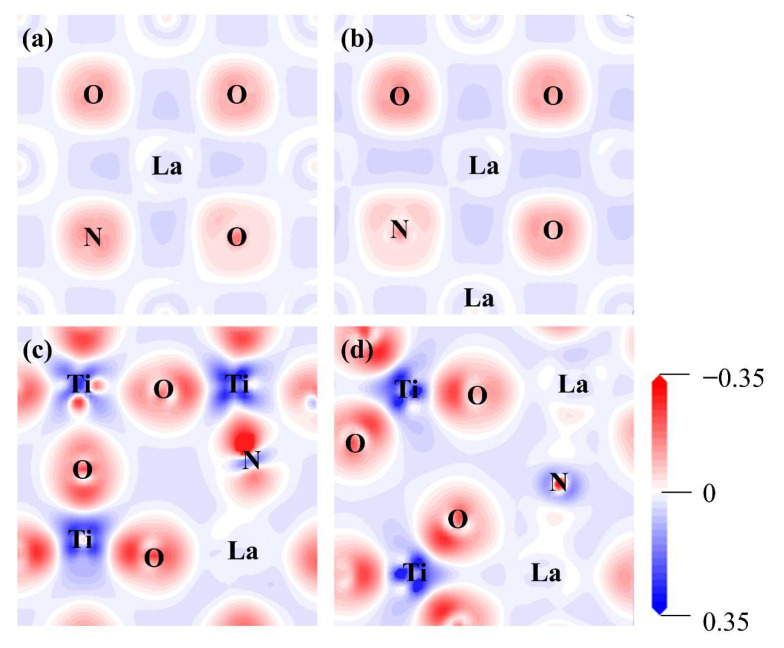
The charge density difference for the (**a**) 12.5% La-N@A co-doped BaTiO_3_, (**b**) 25% La-N@A co-doped BaTiO_3_, (**c**) 12.5% La-N@B co-doped BaTiO_3_, and (**d**) 25% La-N@B co-doped BaTiO_3_. An iso-surface value of 0.1 e/Å^3^ was used, with red and blue clouds indicating the accumulation and depletion of negative charges (electrons), respectively.

A further analysis of the charge density difference of the La-N co-doped BaTiO_3_ models provides a better understanding of how co-doping affects the electronic properties of BaTiO_3_. There was an increase in the accumulation of positive charge around the La atom for the model of La doping at the Ba site compared to the pristine model, as shown in Figure 11a,b. This phenomenon remained almost unaffected by variations in the doping concentration. When La was doped at the Ti site, the charge transfer was significantly increased as compared to it being doped at Ba sites, as shown in Figure 11c,d. Moreover, the introduction of La and N co-doping both influenced the accumulation of positive charges around the Ti atoms, which was significantly increased as compared the pristine model.

The calculated Bader atomic charge transfer values for the La-N co-doped BaTiO_3_ are provided in Table 3, and the detailed breakdown of the Bader atomic charge values for each atom in different models is shown in Table S2 in the Supplementary Materials. The results showed that the amount of charge lost by O^2−^ ions decreased in the La-N@A model as the La concentration was increased. This was consistent with the earlier results obtained from the charge density-difference maps. Meanwhile, the impact of this doping on the charge values of other ions was relatively small. The Bader atomic charge values obtained for La and N did not exhibit any significant variations at different La concentrations. For a 25% doping concentration of La in the co-doped model of La-N@B, the Bader atomic charge for La decreased from 2.12 to 2.05, whereas it decreased from −1.44 to −1.17 for N. This change in the charge distribution can induce a certain degree of lattice deformation, potentially leading to the generation of impurity levels [59], which is in agreement with previous computational results. The calculations also indicated that the Bader atomic charge for La decreased from 2.10 to 2.06 as the La doping concentration increased to 25%, whereas its value increased from −1.45 to −1.09 for N. The increased change in the Bader atomic charge for N may lead to a greater degree of deformation, resulting in the formation of different impurity levels. These findings align with previous computational results.

**Figure 12:**
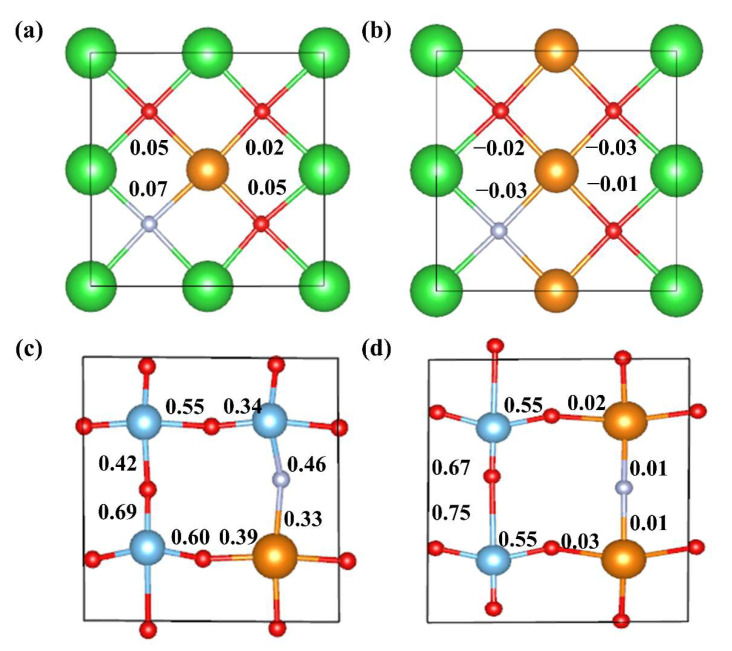
The Mulliken bond populations for the (**a**) 12.5% La-N@A co-doped BaTiO_3_ (1 0 0), (**b**) 25% La-N@A co-doped BaTiO_3_ (1 0 0), (**c**) 12.5% La-N@B co-doped BaTiO_3_ (0 1 0), and (**d**) 25% La-N@B co-doped BaTiO_3_ (0 1 0). Ba atoms are shown in green, Ti atoms in blue, O atoms in red, La atoms in orange, and N atoms in grey.

Mulliken bond populations were calculated for four cut planes depicting typical bonds in co-doped BaTiO_3_ models, as shown in Figure 12. La-N and La-O bond populations are close to zero in the 12.5% La-N@A model, suggesting the formation of ionic bonds. In the 25% La-N@A model, the bond populations of La-N and La-O are small negative values, indicating ionic bonding as well. In addition, in the 12.5% La-N@B model, the bond population for Ti-N is 0.46, indicating a covalent Ti-N bond, while in the 25% La-N@B model, the bond population for La-N is close to zero, indicating an ionic La-N bond.

#### 2.2.3. Optical Properties

The significance of the optical absorption range as one of the most important factors influencing the photocatalytic activities of a photocatalyst cannot be disregarded. Therefore, the optical absorption spectra of the co-doped models of BaTiO_3_ were computed. For a more accurate evaluation of the optical absorption performance, the dielectric function was first converted into the optical absorption function using Equation (6) [60]:

(6) $$\alpha_{\mathrm{abs}}=\sqrt{2}\omega \sqrt{\sqrt{\varepsilon_{1}^{2}\left(\omega \right)+\varepsilon_{2}^{2}\left(\omega \right)}-\varepsilon_{1}\left(\omega \right)}$$

where ω stands for the angular frequency, $\varepsilon_{1}\left(\omega \right)$ is the real part of the complex dielectric constant, and $\varepsilon_{2}\left(\omega \right)$ is the imaginary part of the complex dielectric constant.

As mentioned earlier, a correction to the electronic structure was applied using the GGA + U method, but it did not bring about any notable improvement in the underestimation of the bandgap. This underestimation can introduce significant errors when predicting absorption-related optical properties. Researchers have proposed the use of a “scissor” operator to compensate for such errors [61]. In this study, a scissor operator of 1.20 eV was employed to obtain accurate optical absorption spectra.

**Figure 13:**
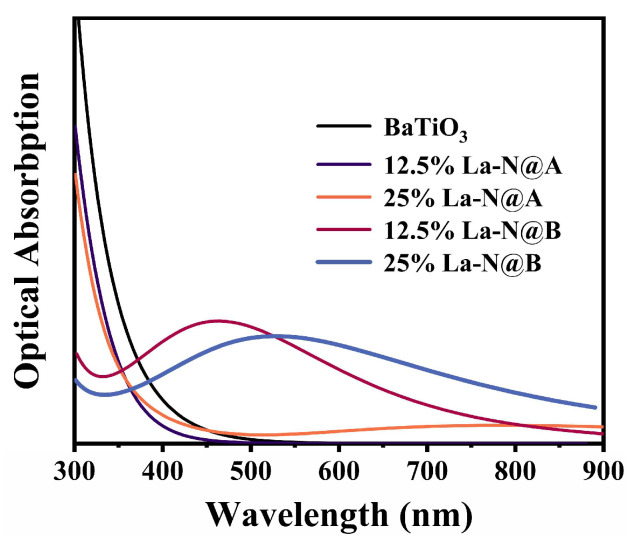
The optical absorption spectra for the pristine and co-doped BaTiO_3_ models.

The computed optical absorption spectra are presented in Figure 13. The absorption curve for pristine BaTiO_3_ aligned well with prior experimental and computational findings [62], with most of the absorption taking place in the ultraviolet region and negligible absorption in the visible light range. A comparison of the absorption curves for BaTiO_3_ structures co-doped with La at the Ba site for the two different concentrations revealed that the 12.5% La-N co-doping did not extend the optical absorption range of BaTiO_3_. Instead, the absorption value dropped to zero at 450 nm, possibly owing to the increased bandgap. Meanwhile, the optical absorption range of BaTiO_3_ stretched beyond 900 nm and exhibited weak absorption around 800 nm as the La doping concentration was increased to 25%. In contrast, the incorporation of La at the Ti site led to a redshift in the absorption curve for both the 12.5% La-N and the 25% La-N co-doped BaTiO_3_ models. The absorption peaks for the two concentrations appeared at 460 nm and 530 nm, respectively. These findings can be attributed to the introduction of an intermediate band, which facilitated the excitation of electrons to the CB. The position of new absorption peaks correlated with the maximum value of the intermediate bandgap, and the closer it was to the CBM, the closer the peak was to the longer wavelengths. At the same time, the main reason limiting the light absorption capacity of the 25% La-N co-doped BaTiO_3_ model was its unwanted wide intermediate bandgap. This is because electrons with lower energy were unable to reach the intermediate band to undergo the second-stage transition. Additionally, it was observed that the absorption edge for the 25% La-N@A BaTiO_3_ and the 25% La-N@B BaTiO_3_ models extended well beyond 1200 nm, possibly indicating metallic trends in these two models. Hence, the results provide ample proof that the optical absorption performance of the BaTiO_3_ photocatalyst can be enhanced significantly by co-doping at the Ti site. However, the DFT cannot accurately describe excited states [63], thus the GW approximation (GWA) or Bethe–Salpeter Equation (BSE) methods have the potential to provide more accurate quantitative descriptions of absorption spectra and optical conductivity [64,65]. In spite of limitations, such DFT calculations can still be used as an approximation for comparison purposes between different BaTiO_3_ doping strategies [11,29,39].

**Figure 14:**
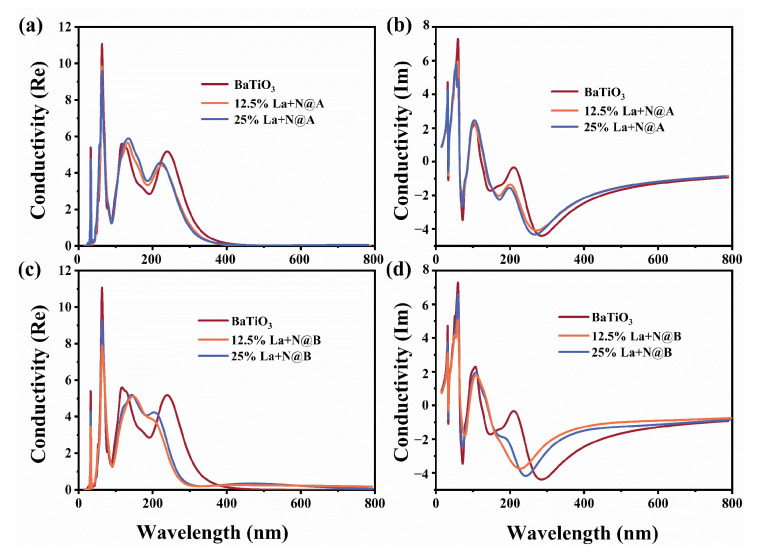
The conductivity plots computed for the (**a**) 12.5% La-N@A co-doped BaTiO_3_, (**b**) 25% La-N@A co-doped BaTiO_3_, (**c**) 12.5% La-N@B co-doped BaTiO_3_, and (**d**) 25% La-N@B co-doped BaTiO_3_.

The optical conductivity of the structures was then calculated to gain a better understanding of the underlying mechanism behind the conduction of photoelectrons [66]. The real part of the conductivity corresponds to actual energy dissipation, while the imaginary part represents the conversion of energy from the electric field to the electronic kinetic energy. The plots displaying the variation in the computed values of conductivity as a function of wavelength for the co-doped models are presented in Figure 14. The real and imaginary components of the conductivity for the La-N@A co-doped BaTiO_3_ model are shown in Figure 14a,b, respectively. On the other hand, the real and imaginary contributions of conductivity in the La-N@B co-doped BaTiO_3_ model are shown in Figure 14c,d, respectively.

The variation in the real part of the conductivity was consistent with the trend followed by the optical absorption function. There was no significant variation in the conductivity for different La doping positions and concentrations at wavelengths below 200 nm, except for changes in the peak magnitudes. For the BaTiO_3_ model co-doped with La at the Ba site, there was a tendency for the last peak to shift to the left upon doping, in both the real and the imaginary parts, accompanied by a decrease in the peak amplitude. However, the influence of the doping concentration on the peak position and value was relatively small. Conversely, a new peak appeared in the real part of conductivity between 300 nm and 800 nm for the BaTiO_3_ model co-doped with La at the Ti site, quite consistent with the results obtained in the absorption spectrum. This verified the photoelectrical performances of the co-doping models in the visible light region. The real part of conductivity for the pristine BaTiO_3_ structure exhibited two peaks within the 100–300 nm range, with the second peak seemingly disappearing in the 12.5% La-N@A model and reappearing with a much smaller magnitude as the concentration of La was increased to 25%. The imaginary part diagram of the conductivity for pristine BaTiO_3_ shows a peak at 200 nm, but the peak value decreases to nearly zero after the co-doping. In the imaginary part, the negative peak shifted to the left and increased in value and concentration.

In spite of the fact that a GGA + U method was used to calculate the electronic structures of the BaTiO_3_ models in order to minimize the underestimations of the bandgap calculated using the PBE functional, the underestimations still exist. Thus, two additional methods were used to calculate the bandgap of the pristine BaTiO_3_ model and compare it to its experimental value. One way is to use the Tauc equation to determine the bandgap values based on our simulated optical absorption spectrum of pristine BaTiO_3_ in Figure 13 [67], and the Tauc curve for pristine BaTiO_3_ is shown in Figure S1 in the Supplementary Materials. It is also possible to estimate the bandgap value from the energy difference between the HOMO and LUMO energy levels. Table 4 presents the bandgap values for BaTiO_3_ obtained from these three methods.

In Table 4, “CBM-VBM” represents the bandgap value obtained by subtracting the valence band maximum (VBM) from the conduction band minimum (CBM) in the band structure plot. “HOMO-LOMO” denotes the bandgap calculated by subtracting the energy of the highest occupied molecular orbital (HOMO) from the lowest unoccupied molecular orbital (LOMO). “Tauc plot” indicates the bandgap value approximated using the Tauc equation based on the optical absorption spectrum of pristine BaTiO_3_ in Figure 14. The bandgap experimental value of pristine BaTiO_3_ is 3.2 eV [68]. According to Table 4, the “HOMO-LOMO” method yields a slightly larger bandgap value than the “CBM-VBM” method, but it is still approximately 0.9 eV lower than the experimental value. Additionally, the bandgap of the pristine BaTiO_3_ approximated by the Tauc equation is 3.79 eV, which is slightly higher than the experimental value of 3.2 eV. This discrepancy may be attributed to errors in the UV-vis spectra in our simulations, which may affect the accuracy of the Tauc equation-derived bandgap values. In general, the bandgap values calculated by the Tauc equation based on the simulated optical absorption spectra appear to be more accurate than those derived from the two other methods.

#### 2.2.4. Water Redox Potential

**Figure 15:**
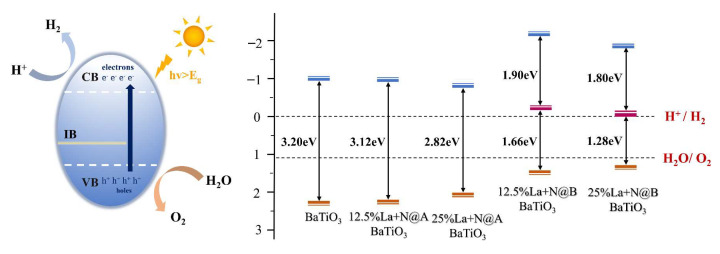
Band edge arrangement of pristine BaTiO_3_ and co-doped BaTiO_3_ models with regard to the potential at the edge of the water redox zone.

Similarly, Equation (3) was employed to calculate the values of *E*_CB_ and *E*_VB_ for the La-N co-doped models. The obtained data are visualized in Figure 15. Compared to the pristine BaTiO_3_ model, all the La-N co-doped models displayed a reduction in the bandgap energy to a certain extent. The band edge positions in the solar-driven water-splitting process adhere to the thermodynamic requirements for hydrogen production, spanning the redox potentials of water. It is known that increasing the capacity to oxidize or reduce water is dependent upon VBM shifting downwards and CBM moving upwards [69]. A comparison of the CBM and VBM positions for pristine BaTiO_3_ and the co-doped BaTiO_3_ models revealed that these positions in the La-N@B co-doped BaTiO_3_ structure were closer to the redox levels of water in comparison to the La-N@A co-doped BaTiO_3_ model. This suggested that the redox capability of the La-N@B co-doped BaTiO_3_ model was superior to that of the La-N@A co-doped BaTiO_3_ model. In the case of La-N@B doping, the positions of the CBM and VBM further decreased and increased, respectively, with an increase in the La concentration. This indicated an enhancement in the redox capability and a shift in the absorption edge into the visible light range. This also explained the absorption edge of the 25% La-N@B doping model being longer than that of the 12.5% La-N@B doping model.

Electrons are energized in the presence of sunshine and move from the VB to the CB. The photocatalytic activity can be enhanced in the presence of intermediate energy levels, since they lower the energy needed for electronic transitions. Moreover, electronic transitions from the VB to the intermediate band are possible only within a reduced energy range facilitated by the appearance of impurity levels in the La-N@B co-doped model. This doping approach leads to the formation of intermediate energy levels, influencing the positions of the CBM and VBM through these intermediate levels. Combined with the earlier findings, the 25% La-N@B co-doped BaTiO_3_ model demonstrated that band edge positions were more favorable for photocatalytic water decomposition.

## 3. Calculation Methods

All our DFT computations were performed using the CASTEP software package in Material Studio [70]. The electronic interactions were modeled using the Perdew–Burke–Ernzerhof (PBE) exchange–correlation functional and the spin-polarized Generalized Gradient Approximation (GGA) functional [71]. The plane-wave basis set was truncated at an energy cutoff value of 500 eV, and a 3 × 3 × 3 k point grid was used for sampling the Brillouin zone. Using the ultra-soft pseudopotential, interactions between the ionic nuclei and valence electrons were described. A GGA + U approach was used to calculate the electronic structures of our co-doped BaTiO_3_ models, with U set at 4.3 eV for Ti-3d and 8.1 eV for La-4f states to minimize the underestimations of the bandgap calculated using the PBE functional [68,72]. The energy convergence was set to 1.0 × 10^−5^ eV/atom, with the maximum force on the atoms equal to 3.0 × 10^−2^ eV/Å, maximum stress on the atoms to 5.0 × 10^−2^ GPa, and maximum atomic displacements to 1.0 × 10^−3^ Å, respectively.

**Figure 16:**
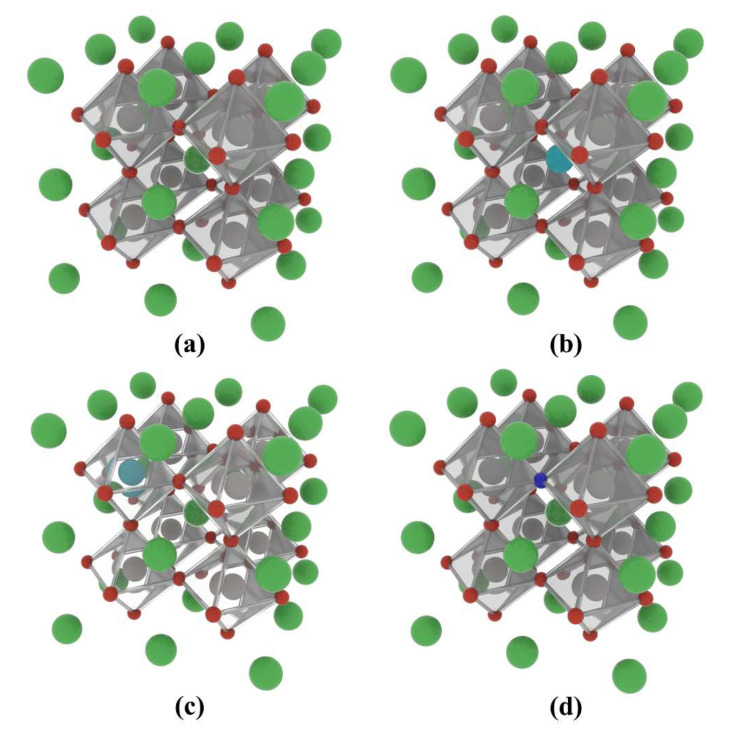
Mono-doped atomic models of the BaTiO_3_ crystal based a 2 × 2 × 2 supercell: (**a**) pristine BaTiO_3_, (**b**) La@A mono-doping, (**c**) La@B mono-doping, and (**d**) N@O mono-doping. Ba atoms are shown in green, Ti atoms in grey, O atoms in red, La atoms in mint blue, and N atoms in dark blue.

In this study, the cubic phase of BaTiO_3_ was studied, with its crystal structure corresponding to the space group of Pm$\overset{-}{3}$m [73,74]. Even though the cubic phase structure is only stable at high temperatures, DFT calculations based on such cubic structures are still a rational and common method for investigating BaTiO_3_’s electronic and optical properties, since DTF calculations based on static configurations do not take temperature influences into account [75,76,77]. The non-metallic element N took over the place of the lattice O atoms, while the rare earth element La was doped into the Ba and Ti atomic positions, with doping percentages of 12.5% and 25%. A 2 × 2 × 2 supercell containing 40 atoms was simulated to investigate the effects of co-doping by the rare-earth element La and the non-metallic element N on BaTiO_3_, as shown in Figure 16. The pristine model of BaTiO_3_ (presented as pristine BaTiO_3_) was then appropriately modified to explore the microstructural impacts of varying the doping concentrations. It was necessary to analyze the effects of mono-doping in BaTiO_3_ to fully understand the impact of co-doping in subsequent calculations. Three types of mono-doped models were studied, including N substituting O sites (presented as N@O), as well as La substituting the Ba and Ti positions (presented as La@A and La@B, respectively).

## 4. Conclusions

The comparative calculations performed in this work using the GGA-PBE in the CASTEP module aimed to examine the structural, electronic, and photocatalytic properties of the BaTiO_3_ structure co-doped with the rare earth element La and the non-metal element N at different positions (Ba and Ti). The energy calculation results indicated that the La-N@A co-doped BaTiO_3_ model exhibited a lower formation and a positive binding energy, theoretically making this doping method more viable to achieve. The addition of an intermediate energy state lowered the energy required for electronic transitions in the La-N@B co-doped BaTiO_3_ model, which considerably modified its band structure and enhanced its performance in the visible light region. Further confirmation of impurity states that were generated by co-doping was provided by calculating the DOS and values of the charge density difference. In the 12.5% La-N@B co-doped BaTiO_3_ model, the N-2p and O-2p orbitals underwent hybridization to generate intermediate states, while the N-2p orbitals dominated the composition of the intermediate states within the 25% La-N@B co-doped BaTiO_3_ model. In terms of the optical properties, the Ti-site co-doping models at both concentrations exhibited widened absorption edges in comparison to pristine BaTiO_3_, resulting in absorption peaks extending into the visible light region, thereby effectively improving the overall optical performance. At the same time, both doping schemes successfully modified the band edge positions in BaTiO_3_, thus improving its capacity to oxidize and reduce water. The findings of this study provide sufficient evidence that the co-doping strategy of La at the Ti sites in BaTiO_3_ with a doping concentration of 25% holds great promise for appropriately tuning the electronic structure of this perovskite material, thus improving the visible light absorption intensity and enhancing its photocatalytic performance.

## Figures and Tables

**Table 1:** Bader atomic charges for mono-doped models.

Model	Ba	Ti	O	La	N
OPT	1.57	2.17	−1.25	-	-
La@A	1.56	2.10	−1.24	2.14	-
La@B	1.57	2.18	−1.24	2.04	-
N@O	1.57	2.17	−1.25	-	−1.22

**Table 2:** The obtained values for *E*_b_ for the co-doped BaTiO_3_ models.

Model	*E*_b_ (eV)
OPT	-
12.5%La-N@A	1.86
25%La-N@A	1.41
12.5%La-N@B	−15.19
25%La-N@B	−15.59

**Table 3:** Bader atomic charges for co-doped models.

**Model**	**Ba**	**Ti**	**O**	**La**	**N**
OPT	1.57	2.17	−1.25	-	-
12.5%La-N@A	1.57	2.15	−1.26	2.12	−1.44
25%La-N@A	1.57	2.11	−1.12	2.10	−1.45
12.5%La-N@B	1.56	2.14	−1.23	2.05	−1.17
25%La-N@B	1.48	2.18	−1.22	2.06	−1.09

**Table 4:** Comparison of bandgap values calculated using three different methods.

**Calculation Methods**	**Bandgap of Pristine BaTiO_3_ (eV)**
CBM-VBM	2.000
HOMO-LOMO	2.298
Tauc plot	3.790
Experimental measurement [68]	3.200

## Data Availability

All data are available within the manuscript. Additional data will be provided upon request from the corresponding authors.

## Supplementary Materials

The following supporting information can be downloaded at: https://www.mdpi.com/article/10.3390/molecules29102250/s1, Figure S1: The Tauc curve chart for the pristine BaTiO_3_ model; Figure S2: Site indexes for our calculated BaTiO_3_ model.; Table S1: The calculated Bader charges for pristine and mono-doped models; Table S2. The calculated Bader charges for pristine and co-doped models.
