# Supplementary material for: Effects of La-N Co-Doping of BaTiO3 on Its Electron-Optical Properties for Photocatalysis: A DFT Study
Source: Molecules. 2024 May 10;29(10):2250. doi: 10.3390/molecules29102250 (PMC11123909; doi:10.3390/molecules29102250)
Supplement: Supplementary file 1 [file molecules-29-02250-s001.zip › molecules-2983479-supplementary.pdf]

Supplementary Materials: Effects of La-N Co-doping of $BaTiO_3$ on its Electron-Optical Properties for Photocatalysis: A DFT Study

Yang Wang [1,2], Qinyan Zhou[1,2], Qiankai Zhang[1,2], Yuanyang Ren [3,4,*], Kunqi Cui[1,2], Chuanhui Cheng[5,*], Kai Wu[3,4]

1 School of Electronics and Information, Xi'an Polytechnic University, Xi'an 710048, China

2 Xi'an Key Laboratory of Interconnected Sensing and Intelligent Diagnosis for Electrical Equipment, Xi'an Polytechnic University, Xi'an 710048, China

3 State Key Laboratory of Electrical Insulation and Power Equipment, Xi'an Jiaotong University, Xi'an 710049, China

4 Electric Power Research Institute of Yunnan Power Grid Company Ltd., China Southern Power Grid, Kunming 650217, China.

5 Electric Power Research Institute, China Southern Power Grid, Guangzhou 510663, China.

* Correspondence:

Yuanyang Ren, Email: yyrenxjtu@126.com (Y. R.)

Chuanhui Cheng, Email: chengchui1239@163.com (C. C.)

1. The Tauc curve of pristine $BaTiO_3$.

Based on the simulated absorption spectrum, the band gap of pristine $BaTiO_3$ was calculated using Tauc equation, as shown in Figure S1.

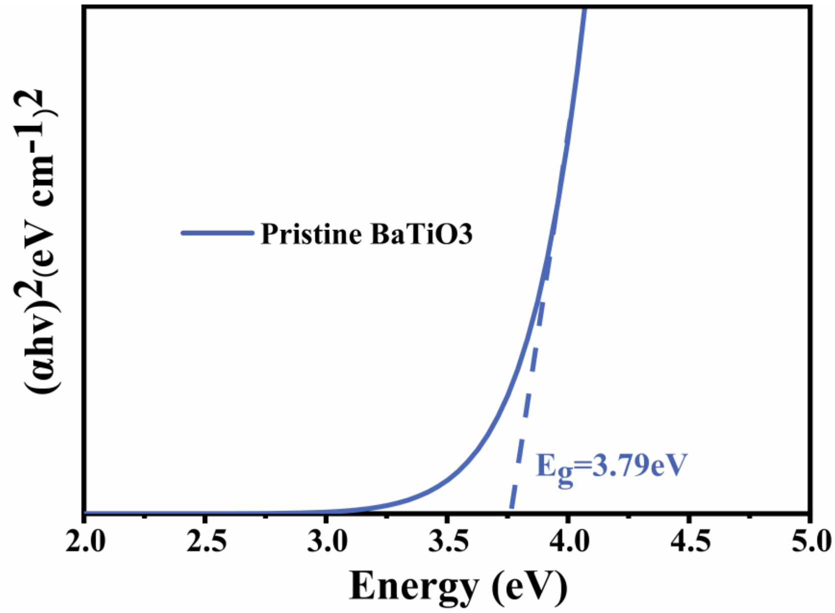

Figure S1. The Tauc curve for pristine model of $BaTiO_3$.

2. The Bader charge analysis and site indexes for $BaTiO_3$ doping models.

The atomic Bader charge for all the $BaTiO_3$ doping models proposed in this paper was calculated. The value of each atom in the mono-doped system is provided in Table S1, while the value of each atom in the La-N co-doping models is shown in Table S2. The Bader charge is calculated by subtracting the total charge from the valence electrons of each atom.

Table S1. The calculated Bader atomic charge for pristine and modo-doping $BaTiO_3$ models.

Model	OPT		La@A		La@B		N@O	
Numbers	Atom	Bader Atomic Charge	Atom	Bader Atomic Charge	Atom	Bader Atomic Charge	Atom	Bader Atomic Charge
1	Ba	1.57	Ba	1.56	Ba	1.57	Ba	1.58
2	Ba	1.57	Ba	1.57	Ba	1.57	Ba	1.58
3	Ba	1.57	Ba	1.57	Ba	1.57	Ba	1.57
4	Ba	1.57	Ba	1.56	Ba	1.57	Ba	1.57
5	Ba	1.57	Ba	1.57	Ba	1.57	Ba	1.57
6	Ba	1.57	Ba	1.56	Ba	1.57	Ba	1.57

7	Ba	1.57	Ba	1.56	Ba	1.57	Ba	1.58
8	Ba	1.57	**La**	2.14	Ba	1.57	Ba	1.58
9	Ti	2.17	Ti	2.10	Ti	2.16	Ti	2.18
10	Ti	2.17	Ti	2.10	Ti	2.18	Ti	2.18
11	Ti	2.17	Ti	2.10	Ti	2.18	Ti	2.14
12	Ti	2.17	Ti	2.10	Ti	2.19	Ti	2.14
13	Ti	2.17	Ti	2.10	Ti	2.18	Ti	2.17
14	Ti	2.17	Ti	2.10	Ti	2.19	Ti	2.17
15	Ti	2.17	Ti	2.10	Ti	2.19	Ti	2.18
16	Ti	2.17	Ti	2.10	**La**	2.04	Ti	2.18
17	O	-1.25	O	-1.25	O	-1.27	O	-1.24
18	O	-1.25	O	-1.25	O	-1.27	O	-1.25
19	O	-1.25	O	-1.25	O	-1.27	O	-1.26
20	O	-1.25	O	-1.25	O	-1.24	O	-1.24
21	O	-1.25	O	-1.25	O	-1.24	O	-1.25
22	O	-1.25	O	-1.24	O	-1.27	O	-1.26
23	O	-1.25	O	-1.24	O	-1.27	O	-1.24
24	O	-1.25	O	-1.25	O	-1.24	O	-1.24
25	O	-1.25	O	-1.25	O	-1.25	O	-1.28
26	O	-1.25	O	-1.24	O	-1.24	O	-1.24
27	O	-1.25	O	-1.25	O	-1.22	O	-1.24
28	O	-1.25	O	-1.24	O	-1.25	O	-1.25
29	O	-1.25	O	-1.25	O	-1.24	O	-1.24
30	O	-1.25	O	-1.24	O	-1.27	O	-1.26
31	O	-1.25	O	-1.25	O	-1.25	O	-1.25
32	O	-1.25	O	-1.25	O	-1.22	O	-1.24
33	O	-1.25	O	-1.24	O	-1.24	O	-1.27
34	O	-1.25	O	-1.24	O	-1.25	O	-1.25
35	O	-1.25	O	-1.24	O	-1.24	O	-1.24
36	O	-1.25	O	-1.24	O	-1.24	O	-1.27
37	O	-1.25	O	-1.25	O	-1.22	O	-1.25
38	O	-1.25	O	-1.24	O	-1.22	O	-1.24
39	O	-1.25	O	-1.24	O	-1.22	O	-1.27
40	O	-1.25	O	-1.24	O	-1.22	**N**	2.04

Table S2. The calculated Bader atomic charge for pristine and co-doping $BaTiO_3$ models.

Model	OPT		12.5%La-N@A		25%La-N@A		12.5%La-N@B		25%La-N@B	
Numbers	Atom	Bader Atomic Charge	Atom	Bader Atomic Charge	Atom	Bader Atomic Charge	Atom	Bader Atomic Charge	Atom	Bader Atomic Charge
1	Ba	1.57	Ba	1.58	Ba	1.57	Ba	1.59	Ba	1.42
2	Ba	1.57	Ba	1.57	Ba	1.56	Ba	1.52	Ba	1.55
3	Ba	1.57	Ba	1.58	Ba	1.57	Ba	1.59	Ba	1.42
4	Ba	1.57	Ba	1.57	Ba	1.57	Ba	1.54	Ba	1.55

5	Ba	1.57	Ba	1.58	Ba	1.56	Ba	1.58	Ba	1.42
6	Ba	1.57	Ba	1.57	Ba	1.57	Ba	1.53	Ba	1.55
7	Ba	1.57	Ba	1.58	**La**	2.10	Ba	1.59	Ba	1.42
8	Ba	1.57	**La**	2.12	**La**	2.10	Ba	1.54	Ba	1.55
9	Ti	2.17	Ti	2.17	Ti	2.13	Ti	2.15	Ti	2.19
10	Ti	2.17	Ti	2.17	Ti	2.13	Ti	2.15	Ti	2.19
11	Ti	2.17	Ti	2.17	Ti	2.12	Ti	2.13	Ti	2.17
12	Ti	2.17	Ti	2.17	Ti	2.12	Ti	2.14	Ti	2.17
13	Ti	2.17	Ti	2.18	Ti	2.12	Ti	2.11	Ti	2.19
14	Ti	2.17	Ti	2.18	Ti	2.12	Ti	2.14	Ti	2.19
15	Ti	2.17	Ti	2.08	Ti	2.06	Ti	2.13	**La**	2.06
16	Ti	2.17	Ti	2.08	Ti	2.06	**La**	2.05	**La**	2.06
17	O	-1.25	O	-1.25	O	-1.26	O	-1.23	O	-1.22
18	O	-1.25	O	-1.25	O	-1.26	O	-1.24	O	-1.27
19	O	-1.25	O	-1.26	O	-1.28	O	-1.16	O	-1.10
20	O	-1.25	O	-1.25	O	-1.26	O	-1.26	O	-1.22
21	O	-1.25	O	-1.25	O	-1.26	O	-1.25	O	-1.28
22	O	-1.25	O	-1.26	O	-1.26	O	-1.26	O	-1.21
23	O	-1.25	O	-1.24	O	-1.24	O	-1.23	O	-1.22
24	O	-1.25	O	-1.25	O	-1.27	O	-1.18	O	-1.22
25	O	-1.25	O	-1.27	O	-1.28	O	-1.21	O	-0.99
26	O	-1.25	O	-1.24	O	-1.24	O	-1.26	O	-1.22
27	O	-1.25	O	-1.25	O	-1.27	O	-1.24	O	-1.22
28	O	-1.25	O	-1.28	O	-1.27	O	-1.28	O	-1.27
29	O	-1.25	O	-1.25	O	-1.25	O	-1.26	O	-1.27
30	O	-1.25	O	-1.24	O	-1.26	O	-1.22	O	-1.13
31	O	-1.25	O	-1.27	O	-1.28	O	-1.24	O	-1.28
32	O	-1.25	O	-1.25	O	-1.25	O	-1.21	O	-1.28
33	O	-1.25	O	-1.24	O	-1.26	O	-1.23	O	-1.28
34	O	-1.25	O	-1.27	O	-1.27	O	-1.27	O	-1.27
35	O	-1.25	O	-1.25	O	-1.24	O	-1.23	O	-1.22
36	O	-1.25	O	-1.25	O	-1.27	O	-1.15	O	-1.10
37	O	-1.25	O	-1.31	O	-1.31	O	-1.25	O	-1.28
38	O	-1.25	O	-1.25	O	-1.24	O	-1.22	O	-1.22
39	O	-1.25	O	-1.25	O	-1.27	O	-1.25	O	-1.21
40	O	-1.25	**N**	-1.44	**N**	-1.45	**N**	-1.17	**N**	-1.09

The site indexes for Tables S1 and S2 are shown in the Figure S2.

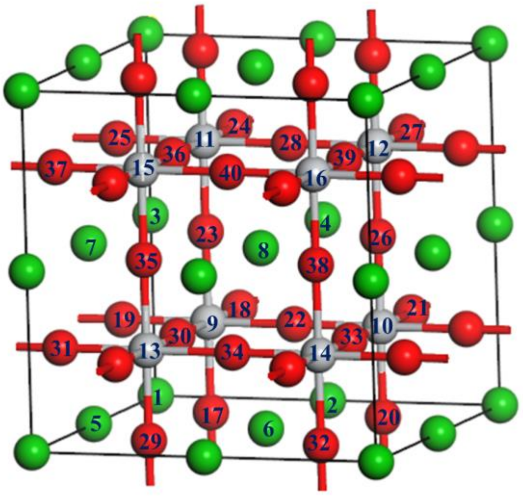

Figure S2. Site indexes for our calculated $BaTiO_3$ models.
